# Supplementary material for: Myeloid cell deficiency of the inflammatory transcription factor Stat4 protects long-term synaptic plasticity from the effects of a high-fat, high-cholesterol diet
Source: Commun Biol. 2023 Oct 2;6:967. doi: 10.1038/s42003-023-05304-0 (PMC10545833; doi:10.1038/s42003-023-05304-0)
Supplement: Supplementary file 2 — Supplementary Material [file 42003_2023_5304_MOESM2_ESM.pdf]

Supplementary Material

Myeloid cell deficiency of the inflammatory transcription factor Stat4 protects long-term synaptic plasticity from the effects of a high-fat, high-cholesterol diet

Xiao-lei Zhang[1], Callie M. Hollander[1], Mohammad Yasir Khan[2], Melinee D'silva[2], Haoqin Ma[1], Xinyuan Yang[1], Robin Bai[3], Coles K. Keeter[3], Elena V. Galkina[3,4], Jerry L. Nadler[2,5], Patric K. Stanton[1]

[1]Department of Cell Biology & Anatomy, New York Medical College, Valhalla, NY 10595, USA.

[2]Department of Pharmacology, New York Medical College, Valhalla, NY 10595, USA.

[3]Department of Microbiology & Molecular Cell Biology, Eastern Virginia Medical School, Norfolk, VA 23507, USA.

[4]Center for Integrative Neuroscience and Inflammatory Diseases, Eastern Virginia Medical School, Norfolk, VA 23507, USA.

[5]ACOS-Research VA Northern California Health Care System, Sacramento, CA 95655, USA.

Supplementary Figures

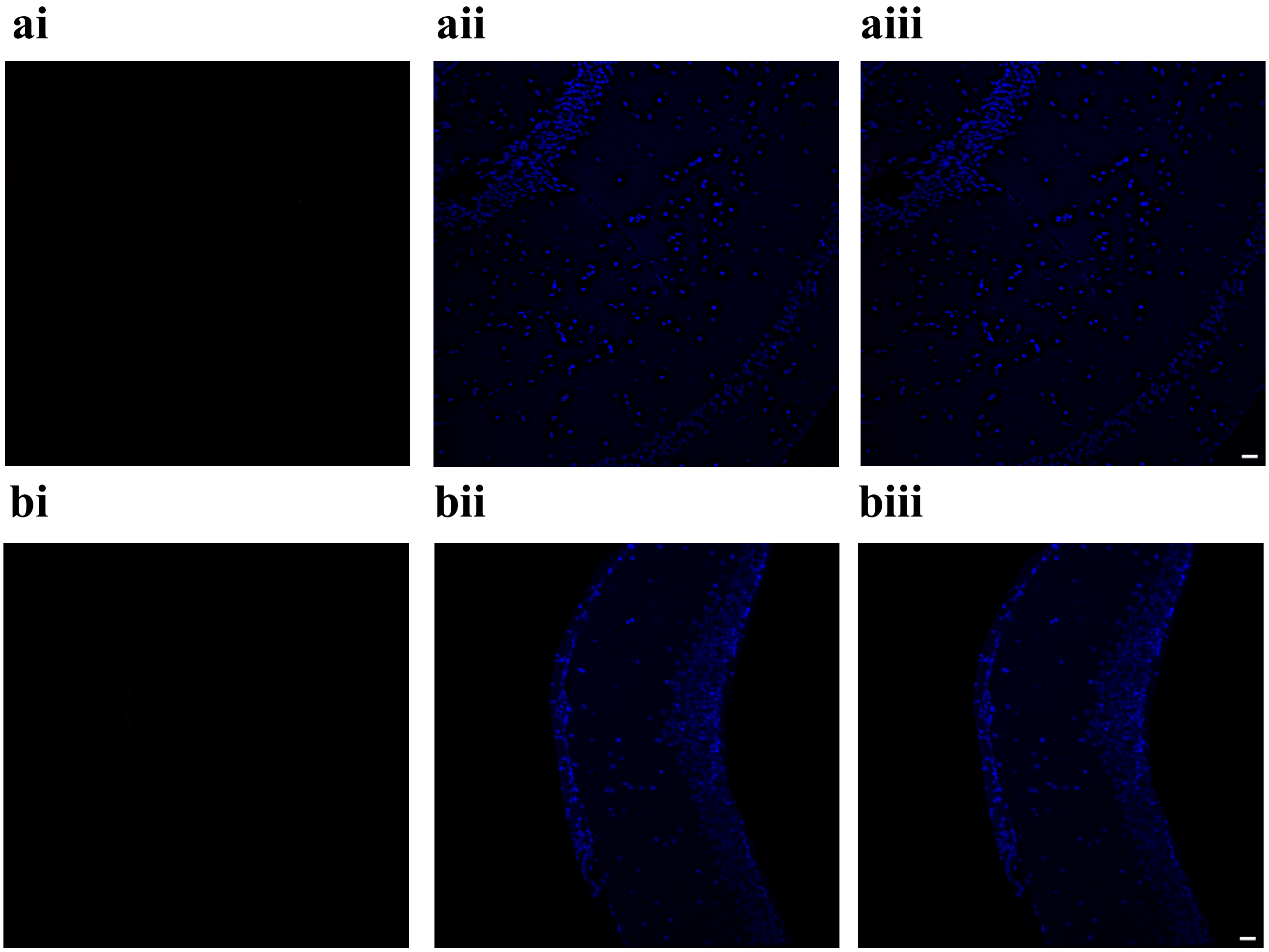

Supplementary Figure 1. Representative negative control immunofluorescence staining in mouse hippocampus stained with only secondary Ab Alexa Fluor 594 (red, **ai, bi**), nuclei stained with DAPI (blue, **aii, bii**), and the two images combined **(aiii, biii)** from **(a)** *Stat4*$^{fl/f}$*Ldlr*$^{-/-}$ and **(b)** *Stat4*$^{fl/f}$*LysM*^{Cre}*Ldlr*$^{-/-}$ mice on DDC diet for 16 weeks (n=4 biologically independent animals per genotype). White scale bars in the lower right of each image indicate 20µm.

Supplementary Figure 2

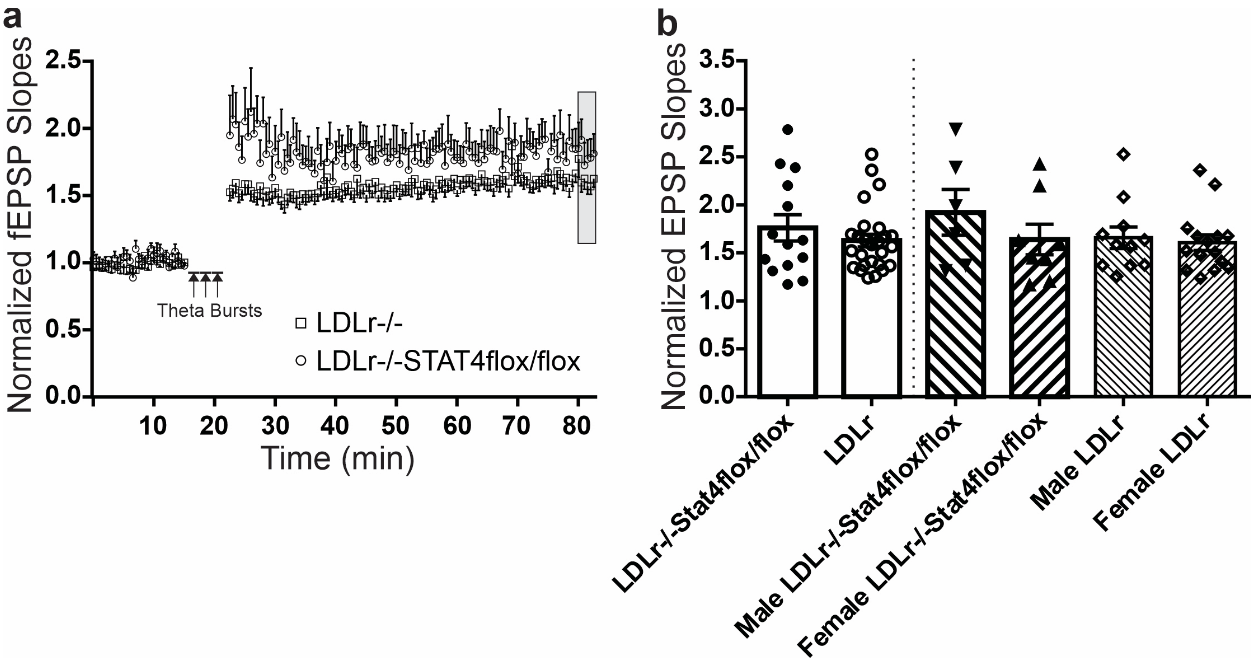

Supplementary Figure 2. LTP at Schaffer collateral-CA1 synapses was not significantly different in *Stat4*$^{fl/f}$*Ldlr*$^{-/-}$ mouse vs. *Ldlr*$^{-/-}$mice. **(a)** Time course of LTP following three theta burst trains of stimulation (TBS x3) of Schaffer collateral axons spaced 3 minutes apart in hippocampal slices from *Stat4*$^{fl/}$*Ldlr*$^{-/-}$ (open circles) and *Ldlr*$^{-/-}$ control mice (open squares) fed a normal chow diet at 3 weeks of age. **(b)** Mean ± SEM magnitude of LTP 60 minute post-TBS, in pooled hippocampal slices from *Stat4*$^{fl/f}$*Ldlr*$^{-/-}$ and *Ldlr*$^{-/-}$ mice, compared to these control mice separated by sex. There were no significant sex differences in the magnitude of LTP in either control mouse strain expressing normal levels of Stat4 when sexes were pooled (Student's t-test, p=0.33), or when separated by sex using two-way ANOVA (F(3,36)=0.74, p=0.54). All error bars are standard error of the mean.

Supplementary Figure 3

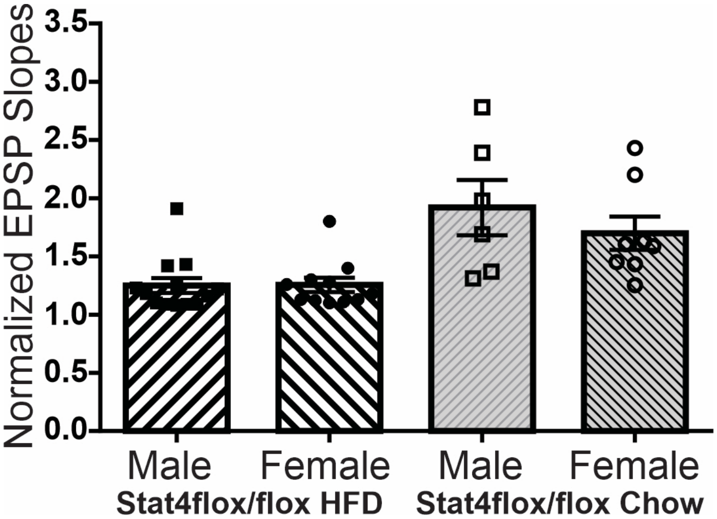

Supplementary Figure 3. LTP at Schaffer collateral-CA1 synapses was significantly affected by diet, but was not significantly different in male vs. female mice fed either chow or HFD-C diet. One-way ANOVA revealed significant differences between the four groups ($F(3,34)=8.025$, $p=0.0004$). Post-hoc Bonferroni multiple comparisons tests showed that the differences resulted from the effects of 16 weeks on the HFD-C diet, which significantly reduced the magnitude of LTP in both male and female *Stat4*$^{fl/f}$*Ldlr*$^{-/-}$ control mice, compared to age-matched 24-25 week old *Stat4*$^{fl/}$*Ldlr*$^{-/-}$ mice fed a chow diet (male HFD-C vs. chow mice, $t=4.01$, $p=0.0019$; female HFD-C vs. chow mice, $t=2.882$, $p=0.0475$). All error bars are standard error of the mean.
