# Supplementary material for: Myeloid cell deficiency of the inflammatory transcription factor Stat4 protects long-term synaptic plasticity from the effects of a high-fat, high-cholesterol diet
Source: Commun Biol. 2023 Oct 2;6:967. doi: 10.1038/s42003-023-05304-0 (PMC10545833; doi:10.1038/s42003-023-05304-0)
Supplement: Supplementary file 3 — Reporting Summary [file 42003_2023_5304_MOESM3_ESM.pdf]

nature portfolio

Corresponding author(s): Patric K. Stanton, Ph.D.

Last updated by author(s): Aug 21, 2023

Reporting Summary

Nature Portfolio wishes to improve the reproducibility of the work that we publish. This form provides structure for consistency and transparency in reporting. For further information on Nature Portfolio policies, see our Editorial Policies and the Editorial Policy Checklist.

Statistics

For all statistical analyses, confirm that the following items are present in the figure legend, table legend, main text, or Methods section.

n/a	Confirmed	
☐	☒	The exact sample size (n) for each experimental group/condition, given as a discrete number and unit of measurement
☐	☒	A statement on whether measurements were taken from distinct samples or whether the same sample was measured repeatedly
☐	☒	The statistical test(s) used AND whether they are one- or two-sided<br>*Only common tests should be described solely by name; describe more complex techniques in the Methods section.*
☐	☒	A description of all covariates tested
☐	☒	A description of any assumptions or corrections, such as tests of normality and adjustment for multiple comparisons
☐	☒	A full description of the statistical parameters including central tendency (e.g. means) or other basic estimates (e.g. regression coefficient) AND variation (e.g. standard deviation) or associated estimates of uncertainty (e.g. confidence intervals)
☐	☒	For null hypothesis testing, the test statistic (e.g. F, t, r) with confidence intervals, effect sizes, degrees of freedom and P value noted<br>*Give P values as exact values whenever suitable.*
☒	☐	For Bayesian analysis, information on the choice of priors and Markov chain Monte Carlo settings
☒	☐	For hierarchical and complex designs, identification of the appropriate level for tests and full reporting of outcomes
☒	☐	Estimates of effect sizes (e.g. Cohen's d, Pearson's r), indicating how they were calculated

Our web collection on statistics for biologists contains articles on many of the points above.

Software and code

Policy information about availability of computer code

Data collection	Data was acquired using SciWorks commercial software from A-M Systems, Inc.
Data analysis	Data was analyzed for fEPSP slope calculation with SciWorks from A-M Systems, Inc. and stististically with GraphPad Prism version 9 and IBM SPSS version 22.

For manuscripts utilizing custom algorithms or software that are central to the research but not yet described in published literature, software must be made available to editors and reviewers. We strongly encourage code deposition in a community repository (e.g. GitHub). See the Nature Portfolio guidelines for submitting code & software for further information.

Data

Policy information about availability of data

All manuscripts must include a data availability statement. This statement should provide the following information, where applicable:

- Accession codes, unique identifiers, or web links for publicly available datasets
- A description of any restrictions on data availability
- For clinical datasets or third party data, please ensure that the statement adheres to our policy

Electrophysiological data are available from FIgshare.com. Nanostring gene expression analysis raw data set is available from NIH GEO. Links to both are supplied in the data availability statement.

Research involving human participants, their data, or biological material

Policy information about studies with human participants or human data. See also policy information about sex, gender (identity/presentation), and sexual orientation and race, ethnicity and racism.

Reporting on sex and gender	N/A
Reporting on race, ethnicity, or other socially relevant groupings	N/A
Population characteristics	N/A
Recruitment	N/A
Ethics oversight	N/A

Note that full information on the approval of the study protocol must also be provided in the manuscript.

Field-specific reporting

Please select the one below that is the best fit for your research. If you are not sure, read the appropriate sections before making your selection.

☒ Life sciences ☐ Behavioural & social sciences ☐ Ecological, evolutionary & environmental sciences

For a reference copy of the document with all sections, see nature.com/documents/nr-reporting-summary-flat.pdf

Life sciences study design

All studies must disclose on these points even when the disclosure is negative.

Sample size	Minimum sample size for each group was selected using power analysis with P preset to P<0.05, to detect parameter differences of 8-10% with a power of 0.85
Data exclusions	Brain slices that did not exhibit stable pre-LTP baselines that did not vary by more than 5% over 15 minutes were excluded from further experimental use. All slices that met this criterion were recorded for at least 90 minutes post-LTP induction, and none were excluded from the data set.
Replication	N/A
Randomization	Animals were assigned to experimental groups by genotype and diet. Whenever possible, slices from control and knockout mice were studied on alternating days.
Blinding	The investigator acquiring the recordings was blinded to the identity of experiment animals until after data acquisition and slope calculations were complete, and the code was broken for assignment to an animal genotype and diet group.

Reporting for specific materials, systems and methods

We require information from authors about some types of materials, experimental systems and methods used in many studies. Here, indicate whether each material, system or method listed is relevant to your study. If you are not sure if a list item applies to your research, read the appropriate section before selecting a response.

Materials & experimental systems

n/a	Involved in the study	
☐	☒	Antibodies
☒	☐	Eukaryotic cell lines
☒	☐	Palaeontology and archaeology
☐	☒	Animals and other organisms
☒	☐	Clinical data
☒	☐	Dual use research of concern
☒	☐	Plants

Methods

n/a	Involved in the study	
☒	☐	ChIP-seq
☒	☐	Flow cytometry
☒	☐	MRI-based neuroimaging

Antibodies

Antibodies used	1) Phospho-STAT4 antibody, Santa Cruz Biotechnology Cat. # 28296<br>2) Alexa Fluor 594 goat anti-mouse IgG, Thermo-Fisher Scientific Cat. # A11005
Validation	1) p-Stat4 Antibody (E-2) is an IgG1 κ mouse monoclonal p-Stat4 antibody that detects Ser 721 phosphorylated Stat4 of mouse, rat and human origin by WB, IP, IF, IHC(P) and ELISA.<br>2) To minimize cross-reactivity, these goat anti-mouse IgG whole antibodies have been cross-adsorbed against human IgG and human serum prior to conjugation. Cross-adsorption or pre-adsorption is a purification step to increase specificity of the antibody resulting in higher sensitivity and less background staining. The secondary antibody solution is passed through a column matrix containing immobilized serum proteins from potentially cross-reactive species. Only the nonspecific-binding secondary antibodies are captured in the column, and the highly specific secondaries flow through.

Animals and other research organisms

Policy information about studies involving animals; ARRIVE guidelines recommended for reporting animal research, and Sex and Gender in Research

Laboratory animals	STAT4-deficient Ldlr-/- mice were genereated by breeding Stat4-/- mice with Ldlr-/- mice (JAX Labs, B6.129S7-Ldlrtm1Her/J). Stat4fl/fl LysMCre Ldlr-/- were generated via cross of newly-generated Stat4fl/fl mice with LysMCre and Ldlr-/- mice in our laboratories.
Wild animals	N/A
Reporting on sex	Sex was recorded for all animals, and after analysis showed no statistically significant sex-differences in the measured parameters, animals were pooled for final analysis and reporting.
Field-collected samples	N/A
Ethics oversight	New York Medical College Institutional Animal Care and Use Committee

Note that full information on the approval of the study protocol must also be provided in the manuscript.
